# Clinical Outcome of Patients with *Escherichia coli* Isolated from Catheter Lumens and/or Peripheral Blood Cultures: A Retrospective Analysis

**DOI:** 10.3390/pathogens13060446

**Published:** 2024-05-24

**Authors:** Álvaro Irigoyen-von-Sierakowski, Marta Díaz-Navarro, Andrés Visedo, Mª Jesús Pérez-Granda, Pablo Martín-Rabadán, Patricia Muñoz, María Guembe

**Affiliations:** 1 Department of Clinical Microbiology and Infectious Diseases, Hospital General Universitario Gregorio Marañón, Dr. Esquerdo, 46, 28007 Madrid, Spain; airigoyenvs@gmail.com (Á.I.-v.-S.); maartadn@gmail.com (M.D.-N.); andresvg777@gmail.com (A.V.); massus@hotmail.es (M.J.P.-G.); pmartinra@salud.madrid.org (P.M.-R.);; 2 Instituto de Investigación Sanitaria Gregorio Marañón, 28007 Madrid, Spain; 3 CIBER Enfermedades Respiratorias-CIBERES (CB06/06/0058), 28029 Madrid, Spain; 4 School of Medicine, Universidad Complutense de Madrid, 28040 Madrid, Spain

**Keywords:** *Escherichia coli*, bacteremia, catheter, lock therapy, differential time to positivity, biofilm, outcome

## Abstract

Background. *Escherichia coli* commonly causes catheter-related bloodstream infection (C-RBSI) in specific populations. The differential time to positivity (DTTP) technique is the recommended conservative procedure for diagnosing C-RBSIs. Methods. We conducted a retrospective study of episodes in which *E. coli* was isolated from catheter lumens obtained using the DTTP technique. Microbiological and clinical data were obtained based on the DTTP technique as either catheter colonization, C-RBSI, or non-C-RBSI. Results. A total of 89 catheter blood cultures were included, classified as follows: catheter colonization, 33.7%; C-RBSI, 9.0%; and non-C-RBSI, 57.3%. Only 15.7% of the catheters were withdrawn, with no positive catheter-tip cultures. We found no statistically significant differences in catheter type, antibiotic treatment, or clinical outcome among the groups, except for the frequency of catheter lock therapy or in the frequency of successful treatment. Mortality was associated with C-RBSI in only one patient. Conclusion. *E. coli* bacteremia diagnosed by the DTTP technique was classified as non-catheter-related in most patients. As the majority of the catheters were retained, *E. coli* bacteremia could not be microbiologically confirmed as catheter-related by the catheter-tip culture. Future studies are needed to assess the profitability of the DTTP technique for diagnosing *E. coli* C-RBSIs.

## 1. Introduction

Staphylococci are the main cause of catheter-related bloodstream infection (C-RBSI). However, the Gram-negative bacillus *Escherichia coli*, which can form biofilms on the catheter surface, remains an important agent in specific populations, including patients with oncologic–hematological conditions and those undergoing hemodialysis [1,2,3,4,5,6,7].

The recommended conservative procedure for the diagnosis of C-RBSI is the differential time to positivity (DTTP) technique, which consists of obtaining blood cultures from catheter lumens and a peripheral vein. The presence of C-RBSI is suspected when the growth of a blood culture obtained from a catheter lumen occurs at least 2 h before the growth of a blood culture obtained from a peripheral vein [8,9]. This process is based on the dispersion of sessile cells from the upper layer of the biofilm on the catheter surface into the bloodstream, causing C-RBSI [9,10]. Therefore, the microbial load from blood obtained through the catheter will be greater. Moreover, the appearance of persistent cells and the detachment of individual cells or microcolonies from biofilms that are in a low metabolically active state can be difficult to diagnose and treat efficiently [11,12]. Nevertheless, the microbiological confirmation of a C-RBSI requires the growth of the same bacteria in the catheter-tip culture as in the blood culture obtained from a peripheral vein. This is rarely achieved, as the catheter is not always withdrawn in the context of a Gram-negative bacterial infection. So, the DTTP technique is recommended as a conservative diagnostic tool which can provide guidance on the origin of bacteremia, but it is not a confirmatory technique by itself, and the results should be interpreted with caution.

In some studies, the presence of high-biofilm-producing strains has been associated with worse clinical outcome [13,14,15,16]. However, there are still controversies, even within the same microorganisms [17,18,19,20].

To our knowledge, there are no reported series describing the microbiological confirmation of suspected *E. coli* C-RBSIs or patient management and outcomes.

## 2. Materials and Methods

This retrospective study was carried out in a 1550-bed tertiary teaching hospital in Madrid (Spain) from 2020 to 2022 and included all infections with *E. coli* isolated either from catheter lumens and peripheral blood cultures or from only catheter lumens with negative peripheral blood cultures, obtained using the DTTP technique. We analyzed microbiological and clinical data, including severity classification systems, such as Charlson score, APACHE II, McCabe 3.

We tested the biofilm production of each strain, as a possible virulence factor associated to worse clinical outcome (which included having C-RBSI or death), with both the crystal violet (CV) assay and the tetrazolium salt (XTT) assay to quantify biomass and metabolic activity, respectively, as previously described [21]. Twenty-four-hour biofilms of *E. coli* strains isolated from blood cultures were formed on the bottom of polystyrene well plates, followed by 3 washes with phosphate-buffered saline and stained separately with both CV and XTT. Experiments were performed in triplicate. The median (IQR) absorbance values for CV and XTT were obtained using a spectrophotometer at 550 nm and 492 nm, respectively [22].

### 2.1. Definitions

Catheter colonization (CC): Positive catheter lumen blood cultures and/or positive catheter-tip culture with a negative peripheral blood culture.

C-RBSI: Positive peripheral- and catheter lumen(s) blood cultures with growth of the same microorganism and a time difference between catheter lumen and peripheral blood culture of ≥2 h and/or positive catheter-tip culture.

Non-C-RBSI: Positive peripheral and catheter lumen(s) blood culture with growth of the same microorganism, and a difference between the catheter lumen and peripheral blood culture growth of <2 h and/or negative catheter culture.

Successful treatment: Catheter maintenance and obtaining sterile control blood cultures.

### 2.2. Statistical Analysis

Qualitative clinical variables are expressed as numbers (percentages) and compared using the chi-square test. Quantitative clinical variables are expressed as the mean (standard deviation) and were compared using the median test. The significance level was set at *p* < 0.05. Comparisons between groups were assessed using the Kruskal–Wallis test, and a *p* value < 0.05 indicated statistical significance. All tests were performed using SPSS Statistics for Windows, v.21.0 (IBM Corp, Armonk, New York, NY, USA).

## 3. Results

We included 89 catheter blood cultures from 81 patients, classified as follows: CC, 30 (33.7%); C-RSBI, 8 (9.0%); and non–C-RBSI, 51 (57.3%). There were eight patients with two different blood culture extractions separated by at least 2 days. The percentage of catheter withdrawals was 15.7%. No positive catheter-tip culture results were recorded; therefore, most episodes could be classified based only on DTTP criteria. Only 1 of the 14 withdrawn catheters was from the C-RBSI group (sent for culture 4 days after DTTP blood was taken), and all 14 yielded a negative culture, which may be explained because all patients were under systemic antimicrobial therapy. Almost half of the patients had oncologic–hematological disease, and 40.4% had an infection at another site. No statistically significant differences were found in catheter type, antibiotic treatment, or clinical outcome between the groups, except for the catheter lock therapy rate, which was greater in the colonization and C-RBSI groups, or in the treatment success rate, which was greater in the non-C-RBSI group (Table 1). C-RBSI-associated mortality was recorded for only one patient who developed septic shock.

As expected, in patients in whom infection was detected at another site (*n* = 36), most *E. coli* infections were non-C-RBSI (75%), 16.7% were CC, and only 8.3% were C-RBSIs. However, among the 53 patients in whom the only presumed source of infection was the catheter, 48 (90.6%) were classified as either CC (*n* = 24) or non-CRBSIs (*n* = 24), and only 5 (9.4%) were classified as C-RBSIs. In instances of *E. coli* non-C-RBSI with no other suspected site of infection, catheter-tip cultures were conducted in only 3 out of 24 patients (12.5%), all yielding negative results. In the remaining 21 cases, the catheters were retained. The mean (SD) duration of antibiotic therapy before catheter removal in these three patients who had their catheter removed was 4.67 (5.51) days. Out of the 24 patients with non-CRBSI, 16 (66.7%) had febrile neutropenia, with a median (IQR) absolute neutrophil count of 0/microliter (0–0).

Regarding biofilm production, the overall medians (IQR) for CV and XTT absorbance were 0.075 (0.000–0.996) and 0.156 (0.051–0.732), respectively. No statistically significant differences in biofilm production were observed between groups. In addition, the median (IQR) for CV and XTT absorbance in the 16 patients who died was 0.055 (0.035–0.170) and 0.159 (0.095–0.265) vs. 0.077 (0.042–0.148) and 0.153 (0.112–0.299) in the 73 alive patients. Therefore, no association was found between biomass (CV) or metabolic activity (XTT) and mortality (CV, *p* = 0.739; XTT, *p* = 0.465).

## 4. Discussion

*E. coli* bacteremia diagnosed by peripheral blood cultures obtained through the DTTP technique were not related to the catheter in most patients. However, most cases could not be microbiologically confirmed or ruled out as catheter-related by catheter culture. Although the mortality associated with *E. coli* C-RBSI was low, treatment was successful in only 37.5% of patients.

The incidence of C-RBSI caused by Gram-negative bacilli [2,7], while still low, has increased in recent years [5,6,23], mainly among oncologic patients and those undergoing hemodialysis [1,2,3,4].

DTTP is currently the recommended technique for diagnosing C-RBSI before catheter removal [8,23,24,25,26], based on the role of biofilm dispersion in the pathogenesis and dissemination of biofilm-associated infections [27,28].

In the present study, the DTTP technique detected only five C-RBSI episodes in patients with *E. coli* bacteremia who exhibited no signs of infection at another site (*n* = 29). This might indicate that, in the remaining 24 episodes categorized as non-C-RBSI, the DTTP technique could have been insufficient to point the catheter as the source of bacteremia. Comparing the outcomes of the non-C-RBSI group (who had *E. coli* bacteremia without the catheter being the source of infection), who were otherwise comparable in terms of age, pre-existing conditions, and gender distribution, supports this statement. It is important to highlight that patients with a colonized catheter and a negative peripheral blood culture from the CC group (*n* = 30) are also susceptible for having a catheter-related infection even with negative cultures in the peripheral vein.

In many cases, the catheter-tip is not sent for culture to confirm the catheter as the source of bacteremia, as described in our study (only 15.7% of the catheters were sent for culture), or, even it is sent for culture, it yielded negative results because the patients are already under antimicrobial therapy, as we observed in our 14 patients in whom the catheter was sent for culture. Moreover, as the study was retrospective, we also were unable to assess whether the 24 patients with fever and CC were only colonized, may have a C-RBSI with blood cultures not being positive yet, or had infection at another site in addition to the catheter. Therefore, it is important to note that the DTTP technique is a conservative diagnostic tool which can provide guidance on the origin of the bacteremia, but results must always be interpreted in the context of the pathogen detected and the patient’s comorbidities.

Regarding the management of the patients, nearly all were treated with systemic antimicrobial therapy, and its duration was comparable between groups. Patients with C-RBSI received more antimicrobial lock therapy; however, the bacterial clearance rate in the C-RBSI group was significantly lower. 

In relation to biofilm production, no correlation was observed between high-biofilm-producing *E. coli* strains and patient outcome, as previously described by Martínez et al. [29]. In contrast, Zhang et al. reported that biofilm production was an independent risk factor of mortality for cancer patients with *E. coli* bloodstream infections [16]. So, further assessing the role of biofilm production in clinical outcomes is needed.

## 5. Conclusions

Despite the relatively low occurrence of *E. coli* C-RBSI (9.0%), the efficacy of treatment, relying on catheter maintenance, and the attainment of negative blood cultures was only successful in 37.5% of cases. Future investigations, incorporating catheter cultures, are imperative to confirm *E. coli* C-RBSI episodes.

## Figures and Tables

**Table 1:** Patient characteristics and outcomes after isolation of *E. coli* from catheter blood cultures obtained using the differential time-to-positivity technique.

Characteristic	Group, *n* (%)				*p*
	Total 89 (100)	Colonization 30 (33.7)	C-RBSI 8 (9.0)	Non–C-RBSI 51 (57.3)	
Median (IQR) age, years	64.00 (51.50–71.50)	64.00 (45.00–73.00)	66.00 (49.25–72.75)	64.00 (56.00–69.00)	0.475
Male sex	54 (60.7)	18 (60.0)	2 (25.0)	34 (66.7)	0.083
Underlying condition					0.444
Hematologic malignancy	39 (43.8)	14 (46.7)	2 (25.0)	21 (42.0)	
Solid organ tumor	30 (33.7)	7 (23.3)	3 (37.5)	20 (40.0)	
Gastrointestinal disease	6 (6.7)	3 (10.0)	0 (0.0)	3 (6.0)	
Renal disease	3 (3.4)	2 (6.7)	0 (0.0)	1 (2.0)	
Organ transplant	3 (3.4)	1 (3.3)	0 (0.0)	2 (4.0)	
Other	8 (9.0)	3 (10.0)	3 (37.5)	3 (6.0)	
Median (IQR) Charlson score	8.00 (4.00–10.00)	8.00 (2.00–10.25)	8.50 (5.25–10.75)	7.00 (4.00–10.00)	0.770
Median (IQR) APACHE II score	11 (6.25–13.00)	11.00 (6.00–12.50)	13.00 (6.75–22.5)	10.00 (7.00–13.00)	0.195
McCabe 3	60 (67.4)	23 (76.7)	3 (37.5)	34 (66.7)	0.077
Type of catheter					0.286
Non-tunneled CVC	5 (5.6)	3 (10.0)	0 (0.0)	2 (3.9)	
Tunneled CVC (Hickman)	19 (21.3)	9 (30.0)	1 (12.5)	9 (17.6)	
Port	43 (48.3)	11 (36.7)	4 (50.0)	28 (54.9)	
PICC	21 (23.6)	6 (20.0)	3 (37.5)	12 (23.5)	
PVC	1 (1.1)	1 (3.3)	0 (0.0)	0 (0.0)	
Median (IQR) in-hospital stay	21.00 (9.00–39.50)	23.00 (10.25–43.25)	16.00 (13.50–33.50)	18.00 (9.00–46.00)	0.238
Median (IQR) time to positivity of peripheral BC	8.95 (7.48–10.34)	NA	8.13 (7.24–9.54)	9.33 (7.57–10.4)	0.905
Median (IQR) time to positivity of catheter lumen BC	8.93 (7.09–10.40)	9.80 (7.10–11.29)	5.86 (3.60–7.45)	8.93 (7.47–10.11)	0.190
Catheter withdrawal	14 (15.7)	9 (30.0)	1 (12.5)	4 (7.8)	0.03
Catheter lock therapy	29 (32.6)	16 (53.3)	6 (75.0)	7 (13.7)	<0.001
Amikacin	28 (31.5)	15 (93.8)	6 (100)	7 (100)	
Ciprofloxacin	1 (1.1)	1 (6.3)	0 (0.0)	0 (0.0)	
Median (IQR) days of lock therapy	7.00 (4.00–10.00)	6.00 (4.25–8.50)	7.00 (4.00–14.00)	10.00 (7.00–10.00)	0.280
IV antimicrobial therapy	87 (97.8)	29 (96.7)	8 (100)	50 (98.0)	0.835
Median (IQR) days of IV antimicrobial therapy	8.00 (6.00–10.00)	7.50 (5.00–9.25)	7.50 (7.00–9.50)	8.00 (6.00–10.00)	0.724
Median (IQR) DDDs	14.00 (3.00–18.00)	15.50 (3.00–18.25)	9.50 (4.25–15.75)	14.00 (3.00–18.00)	0.894
Treatment success rate ^a^	69 (69.7)	16 (53.3)	3 (37.5)	43 (84.3)	0.002
Catheter as the only presumed site of infection	53 (59.6)	24 (80.0)	5 (62.5)	24 (70.6))	
Infection at another site	36 (40.4)	6 (20)	3 (37.5)	27 (52.9)	0.610
Abdominal	13 (14.6)	1 (3.3)	2 (25.0)	10 (19.6)	
Urinary	13 (14.6)	3 (10.0)	1 (12.5)	9 (17.6)	
Biliary tract	8 (9.0)	2 (6.7)	0 (0.0)	6 (11.8)	
Mucosal	1 (1.1)	0 (0.0)	0 (0.0)	1 (2.0)	
Perianal	1 (1.1)	0 (0.0)	0 (0.0)	1 (2.0)	
Crude mortality rate	16 (18.0)	3 (10.0)	3 (37.5)	10 (19.6)	0.1081
C-RBSI-associated mortality rate	1 (1.1)	0 (0.0)	1 (12.5)	0 (0.0)	0.006
Median (IQR) absorbance for CV assay *	0.075 (0.000–0.996)	0.060 (0.000–0.853)	0.069 (0.036–0.142)	0.094 (0.014–0.996)	0.516
Median (IQR) absorbance for XTT assay *	0.156 (0.051–0.732)	0.171 (0.053–0.534)	0.294 (0.117–0.344)	0.144 (0.051–0.732)	0.228

C-RBSI, catheter-related bloodstream infection; IQR, interquartile range; CVC, central venous catheter; PICC, peripherally inserted central catheter; PVC, peripheral venous catheter; DTTP, differential time to positivity; BC, blood culture; IV, intravenous; DDD, defined daily dose; CV, crystal violet; XTT, tetrazolium salt. ^a^ Successful treatment was defined as catheter maintenance and obtaining sterile control blood cultures. * Absorbance was tested for only 72/89 strains.

## Data Availability

The datasets used and/or analyzed during the current study are available from the corresponding author on reasonable request.

## References

[1] Gunardi W.D., Karuniawati A. (2021). Biofilm-Producing Bacteria and Risk Factors (Gender and Duration of Catheterization) Characterized as Catheter-Associated Biofilm Formation. Int. J. Microbiol..

[2] Calò F., Retamar P., Martínez Pérez-Crespo P.M., Lanz-García J., Sousa A., Goikoetxea J., Reguera-Iglesias J.M., León E., Armiñanzas C., Mantecón M.G. (2020). Catheter-related bloodstream infections: Predictive factors for Gram-negative bacteria aetiology and 30 day mortality in a multicentre prospective cohort. J. Antimicrob. Chemother..

[3] Bouza E., Rojas L., Guembe M., Marín M., Anaya F., Luño J., López J.M., Muñoz P. (2014). Predictive value of superficial cultures to anticipate tunneled hemodialysis catheter-related bloodstream infection. Diagn. Microbiol. Infect. Dis..

[4] Hajji M., Neji M., Agrebi S., Nessira S.B., Hamida F.B., Barbouch S., Harzallah A., Abderrahim E. (2022). Incidence and challenges in management of hemodialysis catheter-related infections. Sci. Rep..

[5] Ruiz-Ruigómez M., Aguado J.M. (2021). Duration of antibiotic therapy in central venous catheter-related bloodstream infection due to Gram-negative bacilli. Curr. Opin. Infect. Dis..

[6] Fares J., Khalil M., Chaftari A.M., Hachem R., Jiang Y., Kantarjian H.M., Raad I.I. (2019). Impact of Catheter Management on Clinical Outcome in Adult Cancer Patients with Gram-Negative Bacteremia. Open Forum Infect. Dis..

[7] Guembe M., Pérez-Granda M.J., Capdevila J.A., Barberán J., Pinilla B., Martín-Rabadán P., Bouza E., NUVE Study Group (2017). Nationwide study on peripheral-venous-catheter-associated-bloodstream infections in internal medicine departments. J. Hosp. Infect..

[8] Mermel L.A., Allon M., Bouza E., Craven D.E., Flynn P., O’Grady N.P., Raad I.I., Rijnders B.J.A., Sherertz R.J., Warren D.K. (2009). Clinical practice guidelines for the diagnosis and management of intravascular catheter-related infection: 2009 Update by the Infectious Diseases Society of America. Clin. Infect. Dis. Off. Publ. Infect. Dis. Soc. Am..

[9] Chaves F., Garnacho-Montero J., Del Pozo J.L., Bouza E., Capdevila J.A., de Cueto M., Ángeles Domínguez M., Esteban J., Fernández-Hidalgo N., Fernández Sampedro M. (2018). Executive summary: Diagnosis and Treatment of Catheter-Related Bloodstream Infection: Clinical Guidelines of the Spanish Society of Clinical Microbiology and Infectious Diseases (SEIMC) and the Spanish Society of Intensive Care Medicine and Coronary Units (SEMICYUC). Enfermedades Infecc. Microbiol. Clin. (Engl. Ed.).

[10] Donlan R.M. (2002). Biofilms: Microbial life on surfaces. Emerg. Infect. Dis..

[11] Di Domenico E.G., Oliva A. (2022). The Current Knowledge on the Pathogenesis of Tissue and Medical Device-Related Biofilm Infections. Microorganisms.

[12] Høiby N., Bjarnsholt T., Moser C., Bassi G.L., Coenye T., Donelli G., Hall-Stoodley L., Holá V., Imbert C., Kirketerp-Møller K. (2015). ESCMID guideline for the diagnosis and treatment of biofilm infections 2014. Clin. Microbiol. Infect. Off. Publ. Eur. Soc. Clin. Microbiol. Infect. Dis..

[13] Ryu S.Y., Baek W.K., Kim H.A. (2017). Association of biofilm production with colonization among clinical isolates of Acinetobacter baumannii. Korean J. Intern. Med..

[14] Rajendran R., Sherry L., Nile C.J., Sherriff A., Johnson E.M., Hanson M.F., Williams C., Munro C.A., Jones B.J., Ramage G. (2016). Biofilm formation is a risk factor for mortality in patients with Candida albicans bloodstream infection-Scotland, 2012–2013. Clin. Microbiol. Infect. Off. Publ. Eur. Soc. Clin. Microbiol. Infect. Dis..

[15] Tumbarello M., Fiori B., Trecarichi E.M., Posteraro P., Losito A.R., De Luca A., Sanguinetti M., Fadda G., Cauda R., Posteraro B. (2012). Risk factors and outcomes of candidemia caused by biofilm-forming isolates in a tertiary care hospital. PLoS ONE.

[16] Zhang Q., Gao H.Y., Li D., Li Z., Qi S.S., Zheng S., Bai C.S., Zhang S.H. (2019). Clinical outcome of *Escherichia coli* bloodstream infection in cancer patients with/without biofilm formation: A single-center retrospective study. Infect. Drug Resist..

[17] Pongrácz J., Benedek K., Juhász E., Iván M., Kristóf K. (2016). In vitro biofilm production of Candida bloodstream isolates: Any association with clinical characteristics?. J. Med. Microbiol..

[18] Barsoumian A.E., Mende K., Sanchez C.J., Jr Beckius M.L., Wenke J.C., Murray C.K., Akers K.S. (2015). Clinical infectious outcomes associated with biofilm-related bacterial infections: A retrospective chart review. BMC Infect. Dis..

[19] Guembe M., Alonso B., Lucio J., Pérez-Granda M.J., Cruces R., Sánchez-Carrillo C., Fernandez-Cruz A., Bouza E. (2018). Biofilm production is not associated with poor clinical outcome in 485 patients with *Staphylococcus aureus* bacteraemia. Clin. Microbiol. Infect. Off. Publ. Eur. Soc. Clin. Microbiol. Infect. Dis..

[20] Guembe M., Guinea J., Marcos-Zambrano L., Fernández-Cruz A., Peláez T., Muñoz P., Bouza E. (2014). Is biofilm production a predictor of catheter-related candidemia?. Med. Mycol..

[21] Peeters E., Nelis H.J., Coenye T. (2008). Comparison of multiple methods for quantification of microbial biofilms grown in microtiter plates. J. Microbiol. Methods.

[22] Vandecandelaere I., Van Acker H., Coenye T. (2016). A Microplate-Based System as In Vitro Model of Biofilm Growth and Quantification. Methods Mol. Biol..

[23] Wiggers J.B., Daneman N. (2020). The culture of follow-up blood cultures. Clin. Microbiol. Infect. Off. Publ. Eur. Soc. Clin. Microbiol. Infect. Dis..

[24] Bouza E., Alcalá L., Muñoz P., Martín-Rabadán P., Guembe M., Rodríguez-Créixems M. (2013). Can microbiologists help to assess catheter involvement in candidaemic patients before removal?. Clin. Microbiol. Infect. Off. Publ. Eur. Soc. Clin. Microbiol. Infect. Dis..

[25] Ruiz-Giardin J.M., Ochoa Chamorro I., Velázquez Ríos L., Jaqueti Aroca J., García Arata M.I., SanMartín López J.V., Santillán M.G. (2019). Blood stream infections associated with central and peripheral venous catheters. BMC Infect. Dis..

[26] Wolcott R. (2021). Biofilm and catheter-related bloodstream infections. Br. J. Nurs. (Mark Allen Publ.).

[27] Hall-Stoodley L., Costerton J.W., Stoodley P. (2004). Bacterial biofilms: From the natural environment to infectious diseases. Nat. Rev. Microbiol..

[28] Penesyan A., Paulsen I.T. (2021). Three faces of biofilms: A microbial lifestyle, a nascent multicellular organism, and an incubator for diversity. npj Biofilms Microbiomes.

[29] Martínez J.A., Soto S., Fabrega A., Almela M., Mensa J., Soriano A., Marco F., de Anta M.T.J., Vila J. (2006). Relationship of phylogenetic background, biofilm production, and time to detection of growth in blood culture vials with clinical variables and prognosis associated with *Escherichia coli* bacteremia. J. Clin. Microbiol..

